# Multi-Modal, Machine Learning-Driven Framework Integrating Multi-Omics for Personalized Chronic Kidney Disease Management

**DOI:** 10.3390/jcm15135213

**Published:** 2026-07-03

**Authors:** Bartosz Rutka, Alicja Danieluk, Natalia Wiewiórska-Krata, Krzysztof Mucha

**Affiliations:** 1 Department of Transplantology, Immunology, Nephrology and Internal Diseases, Medical University of Warsaw, 02-006 Warsaw, Poland; 2 Laboratory of Cellular and Genetic Therapies, Center for Preclinical Research, Medical University of Warsaw, 02-097 Warsaw, Poland; 3 Institute of Biochemistry and Biophysics, Polish Academy of Sciences, 02-106 Warsaw, Poland

**Keywords:** chronic kidney disease, precision medicine, multi-omics, machine learning, glomerulonephritis, biomarkers, personalized therapy

## Abstract

Chronic kidney disease (CKD) has become a global health issue, affecting up to 14% of the population worldwide. Between 1990 and 2021, the number of patients grew from 351 million to almost 674 million, with projections warning that CKD may rank as the fifth leading cause of death globally by 2040. Clinically, CKD stems from a complex mix of etiologies, including lifestyle-driven civilization diseases, such as diabetes, hypertension, or obesity, immune-mediated glomerulonephritides (such as IgA, membranous nephropathy or focal segmental glomerulosclerosis), genetic (such as autosomal-dominant polycystic kidney disease, Fabry disease) and tubulointerstitial diseases, or causes of undetermined etiology. Time to diagnosis and the diagnosis of CKD before end-stage organ failure are crucial; therefore, new methods are actively being developed for early detection of kidney disease. Physicians emphasize the need to evaluate markers of kidney dysfunction faster and more accurately. Modern nephrology relies on multi-omics profiling, encompassing genomics, transcriptomics, proteomics, and metabolomics. Applying these technologies to identify molecular drivers of the disease can yield specific signatures that help clinicians stratify patients and decide on a treatment and follow-up plan. Our review addresses a fundamental transformation reshaping nephrology: the transition from a traditional to precision medicine approach.

## 1. Introduction

Chronic kidney disease (CKD) represents a major and growing challenge in healthcare, with a steadily increasing global burden. Despite variability in historical estimates, the increasing prevalence of CKD has been consistently and well documented. According to a Global Burden of Disease study, the affected population increased from approximately 351 million in 1990 to nearly 674 million by 2021, accounting for more than 8.5% of the global population [1]. However, disease prevalence and related mortality are distributed unevenly across several geographical regions. For example, comprehensive epidemiological surveillance in the United States using National Health and Nutrition Examination Survey (NHANES) data indicates that CKD affects approximately 14% of the adult population [2]. Concurrently, contemporary European data from digital medical systems across several countries estimate the pooled prevalence of CKD to be approximately 10%, though substantial heterogeneity exists between specific countries depending on local diagnostic practices and population demographics [3]. This epidemiological shift has been accompanied by increasing mortality rates. Globally, CKD accounted for approximately 1.53 million deaths in 2021, confirming its status as one of the fastest-growing major causes of mortality [4]. Future forecasts predict a continued increase in the number of patients with CKD, directly related to population aging and the pandemic of civilization diseases [5]. Systematic monitoring and classification of CKD rely on international guidelines, such as those issued by Kidney Disease: Improving Global Outcomes (KDIGO), which defines current standards for early detection and treatment [4].

Based on the comprehensive scope of current nephrological challenges and advancements presented here, this review is organized into seven topical sections. Each section represents a critical step in the evolution from traditional clinical assessment to precision medicine.

## 2. Etiology and Biological Heterogeneity of CKD

The etiology of CKD is complex and multifactorial, but it can be divided into subgroups, including civilization (lifestyle-related), autoimmune, genetically driven or tubulointerstitial, and geographically dependent diseases. Although civilization diseases, such as diabetes mellitus (DM), hypertension (HT), and obesity, are considered major contributors to CKD, the effects of environmental factors (stress, overconsumption, climate change) remain relevant.

According to the latest data, DM affects approximately 537 million adults worldwide, representing more than 10% of the population aged 20–79 years [5]. Forecasts indicate that this number will increase significantly by 2050, possibly reaching 783–853 million. DM is a persistent disease that, if left untreated over the long term, leads to disabling and life-threatening health complications [5]. One of the most serious long-term effects of persistent hyperglycemia is kidney damage (nephropathy) [4]. For this reason, regular screening for early diabetes-related complications, including kidney disease, is critical to prevent the development and progression of hyperglycemia [4]. HT is even more widespread, affecting an estimated 1.28 billion adults aged 30–79 years worldwide [6]. HT is not only a key risk factor for cardiovascular disease but also one of the main drivers of the development and progression of CKD [7]. Sustained systemic HT induces structural damage to the delicate renal microvasculature, consequently compromising the organ’s capacity for effective glomerular filtration [7]. The significance of this link is highlighted by high systolic blood pressure being one of the leading modifiable risk factors contributing to CKD-related mortality globally [7]. Obesity, which has doubled in prevalence since 1990, affecting 1 in 8 people worldwide in 2022, is also a major risk factor for the development and progression of CKD [8]. Compared with individuals with a healthy weight, the risk of incident CKD is approximately 40% higher in individuals classified as overweight and 83% higher in individuals with obesity. Moreover, patients with obesity are two to seven times more likely to develop end-stage kidney disease (ESKD) [9]. These three conditions, which often coexist, lead to damaged blood vessels in the kidneys, which initiates fibrosis and a gradual loss of kidney function [9]. 

CKD may also arise from diverse disease mechanisms that affect various renal compartments, pointing to the necessity for a wider and more biologically informed classification system. Hereditary and monogenic kidney diseases constitute a significant group of CKD etiologies. Autosomal dominant polycystic kidney disease is the most prevalent inherited kidney disorder, characterized by progressive bilateral renal cyst growth, kidney enlargement, tubular epithelial dysfunction, interstitial fibrosis, and gradual loss of kidney function [10]. Alport syndrome, caused by pathogenic variants in *COL4A3*, *COL4A4*, or *COL4A5*, leads to structural abnormalities of the glomerular basement membrane, persistent hematuria or progressive proteinuria, often accompanied by extrarenal manifestations such as sensorineural hearing loss and ocular abnormalities [11]. In addition, autosomal dominant tubulointerstitial kidney disease encompasses a genetically heterogeneous group of disorders, including *UMOD*-, *MUC1*-, *REN*-, and *HNF1B*-related diseases, typically presenting with slowly progressive tubulointerstitial fibrosis, bland urinary sediment, limited proteinuria, and frequently delayed diagnosis until advanced CKD [12]. Similarly, Fabry disease, an X-linked lysosomal storage disorder caused by deficient α-galactosidase A activity, results in glycosphingolipid accumulation in podocytes, tubular epithelial cells, endothelial cells, and vascular smooth muscle cells, causing albuminuria, progressive renal dysfunction, and systemic cardiovascular and neurological involvement [13]. Beyond inherited kidney diseases, congenital, obstructive, and reflux nephropathies also contribute to CKD, particularly when urinary tract malformations, chronic obstruction, vesicoureteral reflux, recurrent urinary tract infections, or renal scarring lead to nephron loss, resulting in renal impairment [14]. Furthermore, chronic tubulointerstitial diseases comprise a diverse group of conditions characterized by tubular epithelial injury, interstitial inflammation, microvascular rarefaction, and fibrosis as dominant pathological processes. These disorders may result from immune-mediated, infectious, obstructive, toxic, metabolic, or environmental agents and often present with relatively modest proteinuria compared to primary glomerular diseases. Drug-induced nephrotoxicity represents another clinically relevant and potentially preventable cause of kidney injury. For example, non-steroidal anti-inflammatory drugs can cause hemodynamically mediated kidney injury, acute interstitial nephritis, or chronic analgesic nephropathy. Calcineurin inhibitors in turn, may induce renal vasoconstriction, arteriolopathy, and chronic interstitial fibrosis. Lithium is associated with chronic tubulointerstitial nephritis and nephrogenic diabetes insipidus, whereas cisplatin and aminoglycosides predominantly cause tubular epithelial toxicity [15,16]. Finally, infection-related kidney injury, including infection-associated glomerulonephritis, recurrent pyelonephritis, HIV-associated nephropathy, hepatitis-associated glomerular disease, and infection-related interstitial nephritis, may lead to CKD, depending on host susceptibility, pathogen characteristics, immune response, and treatment exposure [17].

An increasingly important contributor to CKD heterogeneity is its unknown or non-traditional etiology, which arises in specific geographic, environmental, and occupational contexts. Such entities usually affect populations without conventional risk factors, thereby challenging traditional etiological classifications. Among these, Balkan endemic nephropathy is the most clearly defined and is strongly associated with chronic exposure to aristolochic acid, likely through contaminated grain or flour in rural Balkan regions [18]. The disease is characterized by a chronic tubulointerstitial phenotype, DNA adduct formation, mutational signatures, including TP53 alterations, and a strong association with upper tract urothelial carcinoma [19]. In contrast, Uddanam nephropathy, identified in the Uddanam region of Andhra Pradesh, India [20], and Mesoamerican nephropathy, observed primarily among agricultural communities in Mexico and Central America [21], present more complex etiologies. These nephropathies are believed to result from the interplay of occupational heat stress, recurrent dehydration, agricultural exposures, water quality, lifestyle factors, socioeconomic vulnerability, and individual susceptibility [22]. Genetic studies of Mesoamerican nephropathy have explored candidate susceptibility pathways, including *NOS3* polymorphisms rs2070744, 4b/a, and rs1799983, as well as other potential genetic or epigenetic contributors such as *OPCML*, *NGAL*, and *APOE* [21,23]. Metabolomic analyses have begun to characterize molecular signatures associated with Mesoamerican nephropathy, with urinary profiles indicating increased gut permeability, inflammation, altered energy metabolism, and disturbances in NAD+ related pathways, including an elevated kynurenate/tryptophan ratio in high-risk workers [24]. Overall, Balkan nephropathy serves as a model of toxin-driven, exposome-associated CKD with carcinogenic potential, whereas Uddanam nephropathy and Mesoamerican nephropathy exemplify unresolved multifactorial CKD syndromes. For these latter conditions, integration of exposomic, genomic, epigenomic, metabolomic, occupational, and climate-related data is necessary to identify vulnerable populations, define molecular subphenotypes, and inform locally adapted prevention strategies.

The group of immune-mediated diseases includes diseases in which the immune system attacks the kidney structures, leading to the development of glomerulonephritides (GN). The most common GN include IgA nephropathy (IgAN), membranous nephropathy (MN), focal segmental glomerulosclerosis (FSGS), and lupus nephritis (LN).

IgAN is the most common form of primary GN, occurring in about 2.5 per 100,000 people per year [25]. Its pathogenesis is described by the “four-hit hypothesis,” which involves the production of aberrantly glycosylated immunoglobulin A1 (Gd-IgA1), the formation of autoantibodies against it, and the creation of immune complexes. Deposition of these immune complexes in the glomerular mesangium activates the complement system, primarily via the alternative and lectin pathways, leading to local inflammation, mesangial cell proliferation, and damage to the filtration barrier [26,27]. Targeting specific components of this complement cascade has recently emerged as a highly promising therapeutic strategy for progressive IgAN [28], which clinically manifests most often as hematuria, often following a respiratory tract infection, and varying degrees of proteinuria [25]. Key biomarkers directly reflect this pathogenic process. Elevated serum levels of Gd-IgA1 and anti-Gd-IgA1 autoantibodies correlate with disease activity, including increased hematuria and proteinuria [26]. Thus, the serum IgA/C3 ratio is considered a reliable marker of disease progression in adults. In addition, urinary cytokines and chemokines, such as monocyte chemoattractant protein-1 (MCP-1), epidermal growth factor, growth/differentiation factor-15, and interleukin-6 (IL-6), can serve as non-invasive indicators of renal inflammatory activity [29].

MN has an incidence in adults of approximately 1.2 per 100,000 people per year [30]. In most cases, the disease is caused by autoantibodies directed against the phospholipase A2 receptor (PLA2R) on the surface of podocytes [31]. The binding of these antibodies results in the formation of immune complexes on the outer surface of the glomerular basement membrane; subsequent thickening and damage to the basement membrane leads to massive proteinuria and nephrotic syndrome [30]. In a smaller subset of patients, autoantibodies may target other antigens, such as thrombospondin type 1 domain-containing 7A [31] and neural epidermal growth factor-like 1 protein [32]. The discovery of anti-PLA2R antibodies has revolutionized the diagnosis and monitoring of MN. A serum test for these antibodies has very high specificity (>99%) for primary MN, enabling diagnosis without the need for an invasive kidney biopsy [33]. Furthermore, understanding the genetic architecture of MN, particularly the role of risk alleles in *PLA2R1* and *HLA-DQA1*, significantly strengthens these non-invasive diagnostic capabilities [34]. The titer of anti-PLA2R antibodies correlates directly with disease activity, and monitoring these levels is important for guiding immunosuppressive therapy; a decrease in antibody levels (i.e., immunological remission) typically precedes clinical improvement (e.g., reduction in proteinuria) by several months [33].

FSGS is estimated to have an incidence in adults of 0.2 to 1.8 cases per 100,000 people per year [35]. FSGS is not a single disorder but a histopathological pattern of kidney injury characterized by sclerosis of some segmental parts of the glomeruli [35]. The cause is podocyte injury, which can be genetic, idiopathic (likely related to a circulating plasma factor), or secondary [35]. Clinically, nephrotic syndrome is the dominant feature [36]. Though the search continues for a definitive circulating factor in primary FSGS, with candidates including soluble urokinase plasminogen activator receptor and cardiotrophin-like cytokine factor 1, a major breakthrough has occurred in genetics. Risk variants (G1 and G2) in the apolipoprotein L1 (*APOL1*) gene have been identified as a major cause of FSGS in individuals of African ancestry [37]. Genetic testing for *APOL1* has become a critical diagnostic and prognostic tool. Other emerging biomarkers include urinary markers of tubular injury, such as neutrophil gelatinase-associated lipocalin (NGAL), and oxidative stress (e.g., malondialdehyde), which may reflect the severity of the disease process [36].

LN develops in up to 50% of patients with systemic lupus erythematosus (SLE), a disease that affects an estimated 5 million people worldwide [38]. LN is one of the most severe organ complications of SLE, with circulating autoantibodies and immune complexes deposited in various glomerular structures [38]. This leads to a broad spectrum of damage, ranging from mild inflammation to severe, rapidly progressive GN [39]. The clinical picture can be highly varied and includes proteinuria, hematuria, and acute kidney injury [39]. Monitoring relies on a panel of biomarkers. Traditional serum markers, such as anti-double-stranded DNA (anti-dsDNA) antibody titers and levels of complement components C3 and C4, which decrease during active disease, are part of the standard of care [40]. However, there is growing interest in non-invasive urinary biomarkers that may more accurately reflect intrarenal inflammation [40], including cytokines (e.g., tumor necrosis factor-like weak inducer of apoptosis [TWEAK] and IL-6), chemokines (e.g., MCP-1), and tubular injury markers (e.g., NGAL and kidney injury molecule-1) [39]. Changes in the levels of these urinary markers can predict disease flares and may allow for earlier therapeutic intervention [41].

In the pathogenesis of these diseases, hereditary factors play a key role in both transcription (transcriptome) and protein synthesis (proteome), eventually defining the disease phenotype, specifically its clinical presentation and course [37,38]. Moreover, it seems that the common denominator, despite different primary causes, is oxidative stress [42]. Thus, continuous monitoring of oxidative stress-associated biomarkers could play a pivotal role in the decision-making process for early diagnosis and prognostication [43]. In both metabolic and autoimmune diseases, excessive production of reactive oxygen species leads to kidney cell damage and intensifies inflammation and fibrosis. For example, in LN, the massive deposition of immune complexes triggers a strong inflammatory response rich in reactive oxygen species, whereas in IgAN, mesangial cell proliferation in response to IgA deposits contributes to the local oxidative burden [42]. Genetic susceptibilities, although different across diseases, may also modify the body’s response to oxidative stress [42].

The broad etiological spectrum of CKD supports its classification as a biologically heterogeneous syndrome rather than a single disease entity. CKD develops through diverse, and often overlapping, pathogenic mechanisms that differ in clinical, epidemiological, molecular, genetic, environmental, biomarker, geographic, and therapeutic characteristics. This etiological and biological diversity underscores the need for multimodal and multi-omics approaches to refine CKD classification, identify molecular subphenotypes, improve risk stratification, and support individualized treatment selection. Table 1 illustrates this heterogeneity by summarizing major CKD etiologies according to their dominant pathological mechanisms, genetic and environmental contributors, population-level risk patterns, and relevance to precision nephrology.

Regardless of the etiology, the final outcome of CKD is usually the same: progressive loss of kidney function leading to ESKD [1,4]. The clinical symptoms, prognosis, and methods of renal replacement therapy, such as hemodialysis or kidney transplantation, are very similar at this stage [4]. This raises two key questions in modern nephrology, which are addressed in the following sections.

## 3. Strategies to Accelerate CKD Diagnosis

The key role of accelerating the diagnosis of CKD is to identify patients in risk groups and to detect early biomarkers of kidney damage before irreversible changes occur [49]. One of the fundamental and widely discussed strategies in this regard is urine screening, specifically for albuminuria as a marker of glomerular permeability [4]. However, the value of screening is often questioned, particularly in low-risk populations. In a study evaluating the value of urine screening in a young adult population and the limitations of such protocols, routine screening for isolated proteinuria or hematuria was found to have limited diagnostic value for significant kidney disease [50]. However, the study emphasized that the combination of hematuria and proteinuria is a powerful predictor of parenchymal renal disease. These results indicate that, although mass non-selective screening may be inefficient, if proteinuria is detected, further testing should be performed for hematuria [50], especially in the context of GN. Isolated hematuria might be the first sign of early IgAN, whereas massive proteinuria would more strongly suggest MN or FSGS [30,35]. In LN, the urinary sediment can be highly variable, reflecting the complexity of the immunological process [40].

Furthermore, relying solely on standard serum creatinine measurements can markedly delay diagnosis, as creatinine levels often remain within the normal range until a substantial portion of kidney function has already been lost. To overcome this limitation and genuinely accelerate early detection, contemporary diagnostic strategies increasingly emphasize the incorporation of cystatin C. Cystatin C concentrations are independent of muscle mass, age, and diet-related factors. The addition of cystatin C to assessments of estimated glomerular filtration rate (eGFR) provides significantly more sensitive and rapid identification of early-stage CKD than conventional creatinine-based methods [51].

Albuminuria remains one of the most clinically significant prognostic biomarkers in CKD. In addition to its diagnostic utility, the urinary albumin-to-creatinine ratio independently predicts kidney disease progression, cardiovascular events, and all-cause mortality [4,52]. Its integration with eGFR contributes substantially to current CKD risk stratification. Elevated albuminuria categories are associated with progressively higher risk, even when eGFR is relatively preserved, indicating that albuminuria reflects both glomerular injury and systemic vascular risk [53]. Given, that eGFR calculated using cystatin C enhances its accuracy [54] and improves prediction of mortality, cardiovascular outcomes, and kidney failure across diverse populations [55,56], both albuminuria and cystatin C should be considered essential clinical biomarkers that connect traditional CKD assessment with precision-based risk stratification.

## 4. Methods for Improving Patient Monitoring

Multi-omics techniques, such as genomics, transcriptomics, proteomics, metabolomics, and analysis of the gut microbiome, play a significant role in patient monitoring and therapeutic approaches. They allow a deeper understanding of the pathomechanisms of the disease to be obtained in a specific patient and for making decision to treat or monitor [57]. This dilemma is frequently encountered in clinical practice. For example, in IgAN, a primary challenge is distinguishing patients who are likely to progress to renal failure from patients with a benign disease course. Similarly, distinguishing primary FSGS from its genetic variants is crucial for optimal selection of therapy, as these variants may exhibit divergent responses to standard immunosuppression [35,37]. Multi-omics analyses are instrumental in identifying predictive molecular signatures, such as specific gene expression profiles in kidney tissue from patients with LN or quantification of anti-PLA2R antibodies in MN, which subsequently guide prognostic and therapeutic decisions [33,41].

The principles of personalized medicine are equally applicable to CKD driven by civilization diseases. In this context, precise individual risk stratification is essential. Validated clinical tools, such as the Kidney Failure Risk Equation (KFRE), integrate multiple clinical and laboratory variables to compute a personalized percentage risk of progression to ESKD [58]. The application of this risk score subsequently directs the intensity of clinical interventions, ensuring that high-risk patients receive prompt, aggressive management, whereas low-risk individuals can be monitored safely without unnecessary therapeutic burden [58].

Moreover, expanding the analytical focus to the gut microbiome has highlighted the critical diagnostic value of the gut–kidney axis. Recent longitudinal data demonstrate that CKD induces profound intestinal dysbiosis that directly accelerates the systemic accumulation of gut-derived uremic toxins. Monitoring the dynamic shifts in these specific microbial metabolites delivers a novel, non-invasive biomarker panel that can independently predict a rapid decline in kidney function, consequently offering a measurable target for emerging microbiome-directed clinical interventions [59].

## 5. Patient Monitoring and Disease Trajectory Assessment

Nephrologists constantly struggle to alter the trajectory of CKD alongside complex comorbidities or surgical realities, such as oncological nephrectomies. The primary goal remains keeping patients off dialysis. Personalized medicine, heavily powered by integrated multi-omics, offers the most realistic way forward. Molecular profiling of patients enables clinicians to implement targeted preventive strategies (e.g., screening families for genetic forms of FSGS) and to establish precise, mechanism-based diagnoses (e.g., separating LN subtypes) and customized immunosuppression regimens (e.g., in MN or IgAN).

Defining the cellular mechanisms underlying kidney disease fundamentally reshapes therapeutic strategies and informs more targeted, mechanism-based interventions. The specialty is rapidly moving away from rigid, standard protocols; instead, clinicians are adopting a treat-to-target approach. Therapeutic interventions are initiated and adjusted to achieve predefined biomarker targets rather than relying solely on nonspecific reductions in proteinuria. Management of MN provides a clear illustration of this strategy. Tracking anti-PLA2R titers gives clinicians a real-time dial for immunosuppression [60]. If the antibody levels crash quickly, a patient might finish cyclophosphamide in just 8 weeks. This approach minimizes prolonged exposure to potentially toxic therapies. Conversely, a rise in biomarker titers provides an early signal for timely therapeutic intervention, allowing clinicians to preempt clinical relapse [60]. This transition from bench research to personalized care showcases how targeted molecular diagnostics can successfully replace invasive treatment procedures [61].

FSGS provides a comparable paradigm, particularly in the context of genetic determinants. Patients harboring high-risk *APOL1* variants frequently exhibit poor responsiveness to standard immunosuppressive therapies, reflecting a pathophysiology driven by intrinsic podocyte injury and protein toxicity rather than immune-mediated mechanisms [37]. This is where precision medicine matters the most; for example, drugs such as APOL1 channel inhibitor inaxaplin specifically target this defect [44]. Clinicians prescribe it exclusively for patients with the confirmed genetic variant, which boosts success rates and saves remaining patients from useless toxic immunosuppressive regimens [44].

The therapeutic approach to IgAN remains more empirical. In the absence of a definitive molecular target, reducing proteinuria to <1 g/day remains the primary therapeutic objective, and the clinical decision-making process is heavily guided by this threshold. The initiation of baseline renin–angiotensin–aldosterone system blockade, immunosuppressive therapy, and the introduction of novel agents, such as sodium-glucose cotransporter-2 inhibitors and endothelin receptor antagonists, all have a goal of consistently minimizing proteinuria [62]. Recent longitudinal data further support this approach, demonstrating that the long-term trajectory of proteinuria remains a major predictor of disease progression in both children and adults with IgAN [63].

LN requires a slightly different approach because of the complex underlying autoimmunity. No single biomarker reliably predicts LN flares or long-term outcomes; therefore, effective disease management requires the integration of multiple biomarkers to enable a more comprehensive and accurate assessment of disease activity and prognosis. Nephrologists now evaluate urinary proteins, such as MCP-1 and TWEAK, alongside traditional serum tests, such as anti-dsDNA and complement, to more accurately reflect intrarenal inflammatory activity. Monitoring this expanded biomarker panel enables timely and evidence-based adjustment of immunosuppressive therapy, enhancing both precision and clinical confidence in disease management [41].

The clinical relevance of CKD biomarkers depends on their degree of validation and readiness for clinical implementation. Currently, eGFR, albuminuria or proteinuria, urinary sediment, and selected disease-specific markers are the most established tools for diagnosis, monitoring, and therapeutic decision-making [4]. In contrast, many emerging biomarkers provide valuable mechanistic insights but lack sufficient standardization and validation for routine clinical use. Soluble tumor necrosis factor receptors 1 and 2 (sTNFR1 and sTNFR2) show strong prognostic associations with kidney function decline and kidney failure, particularly in diabetic kidney disease [64]. However, their clinical utility is limited by uncertainties regarding causality, assay harmonization, population-specific thresholds, and their incremental value beyond conventional risk markers [65]. Similarly, Klotho and fibroblast growth factor 23 reflect early disturbances in mineral metabolism, tubular function, vascular injury, and cardiovascular risk in CKD [66]. Their use remains primarily investigational due to analytical variability, biological complexity, and limited evidence supporting biomarker-guided interventions to improve outcomes [67,68]. Trimethylamine N-oxide, a metabolite derived from gut microbiota and associated with intestinal dysbiosis, systemic inflammation, cardiovascular risk, and impaired renal clearance, represents a promising marker of the kidney-gut axis [69]. Its interpretation, however, is complicated by factors such as diet, microbiome composition, renal function, and dialysis or transplantation status [70,71]. Therefore, biomarker-based precision nephrology must advance beyond descriptive association studies toward clinically actionable validation. This includes standardized assays, reproducible cut-off values, longitudinal prediction models, external validation across diverse CKD populations, and demonstration of added value over established clinical parameters [71].

## 6. Multi-Omics Technologies

Precision nephrology cannot progress without omics technologies. By interrogating biological systems across multiple layers, from genomic variation to circulating metabolites, researchers are progressively unraveling the molecular complexity underlying CKD. This multi-layered approach isolates novel pathogenic pathways and allows us to stratify patients using their exact biological profiles [57].

Every omics discipline captures a distinct piece of the disease puzzle. Genomics flags inherited risks while transcriptomics tracks which genes are actively expressed, whereas proteomics and metabolomics detail functional proteins and final metabolic states. Table 2 outlines how these specific technologies apply to CKD populations.

Analysis of a single omics layer in isolation is often insufficient to capture the full biological context, necessitating integrative approaches. The combined application of transcriptomics and proteomics exemplifies this paradigm. Merging these two datasets has revealed just how heavily the complement cascade, coagulation, and JAK/STAT pathways drive disease progression. The entire point of this integration is patient sub-classification. Specifically in IgAN, the comprehensive integration of genomics, epigenomics, transcriptomics, and proteomics has provided unprecedented insights into disease heterogeneity, moving the field closer to identifying targets for highly personalized treatment [78]. Nephrologists require precise molecular signatures to classify patients beyond broad clinical phenotypes. Such stratification underpins the implementation of targeted, individualized therapeutic strategies [71].

At the same time, spatial transcriptomics and single-cell RNA sequencing are replacing older methods. Standard bulk omics require tissue homogenization, and that step ruins any spatial context. Spatial methods skip homogenization and map gene expression onto the intact kidney structure. Investigators can now see localized immune cell activation, map fibrotic niches, and examine ligand–receptor crosstalk in the actual microenvironment [79]. The Kidney Precision Medicine Project relies on these precise tools to build cellular atlases. Layering this spatial data over a standard histology slide reveals treatment targets that regular biopsies fail to show [80].

While -omics technologies are increasingly influencing precision nephrology, their impact on routine CKD management remains inconsistent and necessitates cautious interpretation. Genomics has contributed to clinical practice in specific scenarios, including the diagnosis of monogenic kidney diseases, facilitating family screening, refining transplant-related risk assessment, and avoiding unnecessary or ineffective immunosuppression in genetically determined nephropathies [81,82]. Similarly, selected biomarker-based and machine learning (ML)-supported tools have entered clinical evaluation or regulatory pathways; for example, KidneyIntelX.dkd integrates plasma biomarkers with clinical variables to support risk assessment for progressive kidney function decline in adults with type 2 diabetes and early-stage CKD, although it is not intended as a stand-alone diagnostic or staging tool [83,84]. In contrast, transcriptomics, proteomics, metabolomics, single-cell technologies, spatial omics, and microbiome profiling remain primarily investigational in CKD, despite their potential to clarify disease mechanisms, identify molecular subphenotypes, and define therapeutic targets [71,85].

Integration of multi-layer molecular and clinical data through multi-omics approaches facilitates the identification of CKD subphenotypes, prognostic biomarkers, and therapeutic targets. Nevertheless, translating these findings into clinical practice is limited by high costs, variability in sample processing and storage protocols, analytical and interlaboratory inconsistencies, and the absence of standardized bioinformatic and analytical pipelines. Further challenges include incomplete reproducibility across cohorts, restricted access to kidney tissue, complex bioinformatics, inadequate integration with electronic health records and clinical workflows, and insufficient prospective validation in real-world settings [86].

## 7. Diagnostic Approach Combined with Machine Learning

The combination of vast and detailed datasets generated by multi-omics technologies with clinical data requires high-level computational tools, therefore ML, a core domain of artificial intelligence (AI), is emerging as a key technology in evaluating this complexity [87]. Early applications, including risk prediction, diagnosis, and the personalization of care in nephrology were often described as insufficient to achieve the field’s true potential [88], and recent improvements have positioned AI as an innovative instrument capable of overcoming traditional clinical challenges, moving from theoretical algorithms to practical, individualized risk stratification [87].

In addition to molecular data analysis, ML is transforming renal pathology through digital nephropathology. Deep learning algorithms are now trained on digital images of kidney biopsies to automatically quantify fibrosis, classify glomerular lesions, and predict clinical outcomes. By minimizing subjective interpretation and inter-observer differences, these automated tools enhance objectivity and reproducibility, supporting the expertise of human pathologists and potentially reducing diagnostic variability [89]. Moreover, ML models outperform traditional statistical methods, such as Cox regression, in predicting CKD progression, because they can capture nonlinear and complex interactions among numerous risk factors [87,88].

In this context, Moszczuk et al. [90] demonstrated the application of ML to classify GN subtypes based on urinary osteopontin levels. The developed algorithm achieved 87% accuracy in differentiating IgAN from other forms of GN, demonstrating the power of ML to extract clinically relevant patterns from biological data, even in smaller datasets. This computational approach perfectly complements earlier genetic findings, which identified the complex role of *SPP1* gene polymorphisms and varying urinary osteopontin excretion in the immunomodulation of IgAN [91]. Although this study focused on classification rather than progression, the principle is directly applicable to creating multi-factorial risk prediction models [90].

**Figure 1:**
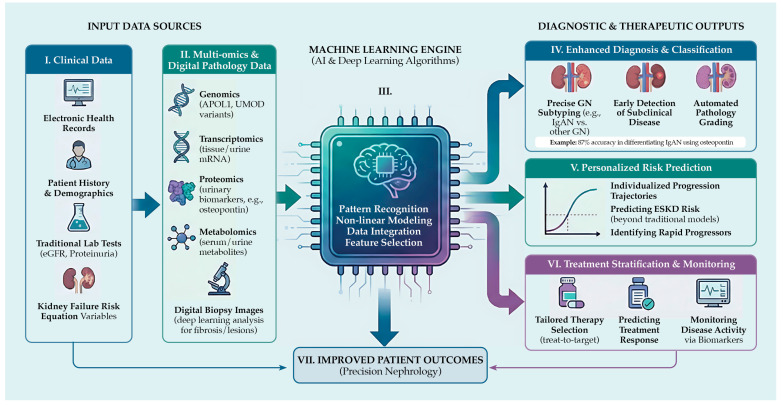
Conceptual overview of a multi-modal, machine learning-driven framework for precision nephrology integrating heterogeneous data sources to improve diagnostic, prognostic, and therapeutic decision-making in chronic kidney disease. Input data sources comprise (I) clinical data, including electronic health records (EHRs), patient history and demographics, traditional laboratory parameters such as estimated glomerular filtration rate (eGFR) and proteinuria, and Kidney Failure Risk Equation (KFRE) variables, as well as (II) multi-omics and digital pathology data, including genomics (e.g., *APOL1* and *UMOD* variants), transcriptomics (tissue and urine mRNA), proteomics (urinary biomarkers, such as osteopontin), metabolomics (serum and urine metabolites), and digital biopsy imaging analyzed using deep-learning-based approaches. These high-dimensional datasets are integrated within a machine learning engine (III) (artificial intelligence and deep learning algorithms), enabling pattern recognition, non-linear modeling, feature selection, and cross-domain data integration. The analytical outputs are translated into clinically actionable domains: (IV) enhanced diagnosis and classification, including precise glomerulonephritis (GN) subtyping and early detection of subclinical disease, as well as automated pathology grading; (V) personalized risk prediction, encompassing individualized disease trajectories, improved estimation of the progression risk beyond conventional models, and identification of rapid progressors; and (VI) treatment stratification and monitoring, supporting tailored therapeutic selection (treat-to-target strategies), prediction of the treatment response, and longitudinal monitoring using validated biomarkers. Collectively, this integrative framework supports improved patient outcomes (VII) by enabling the transition from traditional clinical assessment to precision nephrology.

A summary of ML-driven diagnostic strategies in CKD, from raw data inputs to individualized therapeutic outputs, is presented in Figure 1.

## 8. Benefits and Risks of Machine Learning

The implementation of ML in clinical practice offers considerable clinical benefits, but also has important limitations that warrant careful consideration before widespread adoption. Although early identification of patients at high risk of CKD progression is a major advantage, transitioning to routine practice involves specific technical and ethical barriers. ML approaches offer significant potential to improve risk prediction, patient stratification, image analysis, and the integration of high-dimensional omics data. Nevertheless, many current models lack robust validation, generalizability, interpretability, and proven clinical utility [92,93,94]. The future impact of ML in nephrology will depend on the development of models that are accurate, interpretable, reproducible, fair, externally validated, and demonstrably beneficial for patient care. Achieving these goals will require ongoing collaboration among developers, nephrologists, ethicists, and patients to ensure safe clinical implementation, minimize systemic bias, and prioritize outcomes that are meaningful to patients. A comparative overview of these opportunities and possible pitfalls is provided in Table 3.

## 9. Future Perspectives

Moving precision nephrology forward requires AI and multi-omics translation into prospective clinical practice rather than keeping them confined to retrospective research. To address long-standing challenges in data privacy and computational bias, researchers expect frameworks, such as federated learning, to become standard practice. In contrast to conventional centralized models, federated learning allows ML systems to be trained across multiple institutions without the direct exchange of sensitive electronic health records [98]. This distributed setup protects patient privacy while allowing researchers to include highly heterogeneous demographic data. Ultimately, this leads to the creation of much more robust and unbiased algorithms. In addition, advances in precision medicine will increasingly rely on novel preclinical models, such as 3D bioprinting, which allows for the generation of finely patterned, multicellular tissue architectures to better simulate autoimmune disease microenvironments and accurately test treatment responses [99].

Moreover, the effective integration of predictive systems into the routine clinical workflows necessitates a shift toward rigorous, prospective clinical trials. Currently, most AI models in nephrology are validated retrospectively. Demonstrating their clinical utility requires randomized controlled trials to assess whether ML-guided interventions improve clinical outcomes relative to standard care.

Finally, regulatory systems must evolve to keep pace with rapid technological progress. The widespread adoption of AI tools will depend on compliance with emerging regulatory frameworks for software as a medical device established by agencies such as the US Food and Drug Administration and the European Medicines Agency [100]. Advanced diagnostic strategies in nephrology will transition safely into evidence-based standards of care only amid robust, multi-center, prospective validation and tight regulatory supervision.

## 10. Conclusions

CKD remains a significant global health challenge, underscoring the need to shift from conventional, population-based management strategies to precision nephrology. Disease-specific biological profiling provides substantial clinical value by improving diagnostic accuracy, enabling treat-to-target interventions, and reducing unnecessary therapeutic exposure. The integration of multi-omics data with machine learning offers a robust framework for stratifying patients according to molecular mechanisms, predicting disease progression, and guiding individualized therapeutic decisions. Although challenges persist regarding cost, data harmonization, clinical validation, and regulatory implementation, the integration of multi-omics data with machine learning demonstrates considerable potential to advance personalized CKD management and enhance long-term renal outcomes.

## Figures and Tables

**Table 1:** The heterogeneity of CKD.

CKD Etiology	Main Pathological Mechanism	Genetic & Environmental Contributors	Population Distribution	Clinical Relevance for Precision Nephrology	References
Diabetic kidney disease	Metabolic and hemodynamic injury, hyperfiltration, inflammation, fibrosis	Polygenic T2 DM, obesity, HT, lifestyle-related	High global burden; increased risk in Indigenous, Hispanic, and South Asian populations	Risk stratification through integration of metabolic, vascular, and omics-based markers	Sun et al. [5] Hojs et al. [9]
Hypertensive nephropathy	Chronic vascular injury, ischemic glomerulosclerosis	*APOL1* risk variants, polygenic HT Persistent HT, high salt intake, obesity	Global; significantly higher burden in populations of African ancestry	Integration of Ancestry-Informed Genetic Risk Assessment with Blood Pressure Management	Zhou et al. [6] Brauer et al. [7]
IgA nephropathy	Gd-IgA1 deposition, alternative complement activation	HLA and non-HLA loci. Mucosal infections, microbiome shifts, dietary triggers	Highest prevalence in East Asian populations; frequent in Europe	Biomarker-driven progression prediction (e.g., Gd-IgA1) and targeted therapies	Schena et al. [25] Suzuki et al. [26]
Membranous nephropathy	Autoantibody-mediated podocyte injury (e.g., anti-PLA2R)	*HLA-DQA1*, *PLA2R1* variants. Infections, malignancy, drugs	Worldwide in adults; secondary causes vary geographically	Anti-PLA2R guided diagnosis, monitoring, and treat-to-target immunosuppression	Murtas et al. [33] Xie et al. [34]
Focal segmental glomerulosclerosis	Podocyte injury, segmental glomerular scarring	*APOL1* variants, monogenic podocyte defects. Obesity, viral infections, reduced nephron mass	*APOL1*-associated forms are highly prevalent in African ancestry	Genetic screening for differentiation between immune and genetic forms to inform therapeutic strategies	Rout et al. [35] Egbuna et al. [44]
Lupus nephritis	Immune complex deposition, systemic autoimmunity	Polygenic autoimmune susceptibility. Gender, UV exposure, infections	Higher severity in individuals of African, Hispanic, and Asian ancestry	Integration of urinary biomarkers (e.g., MCP-1) for flare prediction	Parikh et al. [38] Alduraibi et al. [41]
Autosomal-dominant polycystic kidney disease	Cyst formation, tubular epithelial proliferation	*PKD1/PKD2* pathogenic variants HT, nephrotoxic exposure	Worldwide; clear familial clustering	Genotype-informed prognosis guiding surveillance and therapies	Carney et al. [45]
CKD of unknown origin	Chronic tubulointerstitial injury without typical DM/HT presence	Susceptibility variants (e.g., *NOS3*, *APOE*). Heat stress, dehydration, agrochemicals	Agricultural communities (e.g., Mesoamerica, Uddanam, Balkan regions)	Integration of environmental exposure data with metabolic profiling (e.g., altered NAD metabolism)	Marín-Medina et al. [21,23] Raines et al. [24]
Congenital anomalies	Abnormal kidney/urinary tract development, nephron deficit	Monogenic/polygenic developmental variants. Prematurity, urinary obstruction	Global; major cause of pediatric CKD	Early genetic assessment for risk prediction and lifelong surveillance	Murugapoopathy et al. [46]
Infection-related CKD	Glomerular, vascular, or tubulointerstitial injury related to chronic or recurrent infection	Host immune-response variants may modify susceptibility HIV, hepatitis B/C, malaria, schistosomiasis, recurrent pyelonephritis, opportunistic viral infections	Burden varies geographically; higher in regions with endemic infections and limited access to prevention or antiviral therapy	Molecular pathogen profiling and viral load monitoring facilitate the implementation of targeted antimicrobial therapies and the optimization of individualized immunosuppression.	Bonner et al. [47] Khalighi et al. [48]

**Table 2:** Overview of omics technologies and their application in CKD.

Omics Level	Principle of Study	Clinical Application in CKD	Key Outcomes	References
Genomics	Complete set of organism’s DNA (static genetic code).	Identifies genetic predispositions and rare monogenic causes of kidney disease.	• *APOL1* risk variants discovered in African ancestry populations in 2010. • Variants in *UMOD* and *SHROOM3* (key GWAS studies: 2009–2010). • Monogenic diseases: Fabry disease, Alport syndrome (genetic diagnostic development since the 1990s). • Identification of novel *NR3C1* polymorphisms associated with IgAN and MN susceptibility (2021). • GWAS-driven prioritization of drug targets and pathogenic signaling pathways in IgAN (2023).	Egbuna et al. [44] Pac et al. [72] Kiryluk et al. [73]
Transcriptomics	Complete set of RNA transcripts (dynamic gene expression).	Reveals active signaling pathways (inflammation, fibrosis, stress) in tissue biopsies or urine sediment (“liquid biopsy”).	• Identification of active inflammatory pathways in situ (clinical relevance increased with spatial transcriptomics, 2019). • Assessment of cellular stress responses.	Oliverio et al. [74]
Proteomics	Large-scale study of proteins (functional effectors).	Discovers non-invasive biomarkers in body fluids (urine, serum) to predict disease progression.	• CKD273 panel (urinary peptidome) for predicting CKD progression better than albuminuria (2010). • Defining clinical subgroups and predicting treatment responses in pediatric nephrotic syndrome using urine proteomics (2025).	Mihai et al. [75] Cummins et al. [76]
Metabolomics	Complete set of small-molecule metabolites (final phenotype).	Identifies metabolic signatures of impaired function and genome–environment interactions.	• Accumulation of uremic toxins (e.g., indoxyl sulfate), with metabolomic profiling development occurring primarily in the 2010s. • Disturbances in energy metabolism.	Hocher et al. [77]

**Table 3:** Benefits versus risks of implementing machine learning (ML) in nephrology [87,95,96,97].

Potential Benefits	Associated Risks and Challenges
Early and Accurate Risk Prediction: ML algorithms can process high-dimensional data (omics + clinical) to identify rapid progressors earlier than traditional methods.	The ‘Black Box’ Problem: Many high-performance models (e.g., deep learning) lack interpretability. The inability to explain the biological reasoning behind a prediction can undermine clinicians’ trust in the model.
Optimization of Healthcare Resources: Stratifying patients allows expensive or intensive therapies to be directed only to high-risk individuals.	Algorithmic Bias: Models trained on non-diverse populations may perform poorly for underrepresented groups, inadvertently amplifying existing health disparities.
Informed Shared Decision-Making: Providing patients with personalized prognostic data facilitates better understanding and adherence to therapy.	Lack of Generalizability: A model validated in one hospital system may fail in another due to differences in data coding or patient demographics (‘overfitting’), limiting real-world utility.

## Data Availability

No new data were created or analyzed in this study. Data sharing is not applicable.
